# Comparative Analysis of Heart Regeneration: Searching for the Key to Heal the Heart—Part II: Molecular Mechanisms of Cardiac Regeneration

**DOI:** 10.3390/jcdd10090357

**Published:** 2023-08-22

**Authors:** Juan Manuel Castillo-Casas, Sheila Caño-Carrillo, Cristina Sánchez-Fernández, Diego Franco, Estefanía Lozano-Velasco

**Affiliations:** 1 Cardiovascular Development Group, Department of Experimental Biology, University of Jaén, 23071 Jaén, Spain; jmcasas@ujaen.es (J.M.C.-C.); scano@ujaen.es (S.C.-C.); csfernan@ujaen.es (C.S.-F.); dfranco@ujaen.es (D.F.); 2 Medina Foundation, 18007 Granada, Spain

**Keywords:** cardiac disease, myocardial infarction, molecular pathways, hypoxia, metabolism, inflammation, cell cycle, fibrosis, heart regeneration

## Abstract

Cardiovascular diseases are the leading cause of death worldwide, among which ischemic heart disease is the most representative. Myocardial infarction results from occlusion of a coronary artery, which leads to an insufficient blood supply to the myocardium. As it is well known, the massive loss of cardiomyocytes cannot be solved due the limited regenerative ability of the adult mammalian hearts. In contrast, some lower vertebrate species can regenerate the heart after an injury; their study has disclosed some of the involved cell types, molecular mechanisms and signaling pathways during the regenerative process. In this ‘two parts’ review, we discuss the current state-of-the-art of the main response to achieve heart regeneration, where several processes are involved and essential for cardiac regeneration.

## 1. Introduction

Cardiovascular diseases are the leading cause of death worldwide and among all of them, ischemic heart disease affects 1.72% of the world’s population [1,2]. Myocardial Infarction (MI) is driven by the decrease or complete cessation of blood flow to a portion of the myocardium [3] and is characterized by loss of cardiomyocytes (CMs) through different mechanisms of cell death [4]. The damaged contractile myocardium is replaced by myocardial fibrotic tissue, leading to function impairment, which finally ends in heart failure (HF) [5,6,7,8]. One of the major challenges in human cardiovascular research is to find the key for mammalian adult hearts to face the substantial loss of CMs after MI [9,10]. This second part of the review has as its main objective to provide an in-depth analysis summarizing the main molecular targets and related signaling pathways involved in MI and regeneration progression.

## 2. Molecular Bases of Cardiac Regeneration

As previously mentioned, cardiac researchers and clinicians are keen on the identification of the combinatorial molecular response, which enables cell cycle re-entry and division of resident CMs after injury. In this section, we highlight the main molecular effectors needed, within the different steps after injury, which successfully induce and accomplish cardiac regeneration in different animal models.

## 3. The Importance of a Depressed O_2_ Environment for Heart Regeneration

Cardiac damage in MI is typically due to an occlusion of the coronary vessels promoting a drastic reduction of oxygen (O_2_) supply through the blood flow [11]. During the process, the family of hypoxia-inducible factor (HIF) transcription factors are direct effectors of the hypoxic activity [12]. During the initial phase of cardiac ischemia, there are high levels of Hif1α which activate several downstream targets preserving CMs from the hypoxic environment [13]. However, if the hypoxic situation is prolonged, CMs will enter into the apoptotic program, damaging heart tissue and function [13]. As it has been mentioned previously, cardiac regeneration is different among species, even if life stage is considered. Several studies have brought to the forefront a high number of regulators related to cardiac regeneration in vertebrates, and in the last decade, oxygen-related processes, such as environmental hypoxia, hypoxia-induced cellular signaling and mitochondrial metabolism, have appeared among those on the top list [14,15,16,17].

### 3.1. Hypoxic Environment Promotes Cardiomyocytes Proliferation

Reduced levels of oxygenation are predominant in aquatic vs. air environments. In this scenario, fishes, amphibians and mammalian embryos have an oxygen pressure (PaO_2_) in arterial blood of 25–35 mmHg, whereas arterial PaO_2_ in newborn mammals rise up to 100 mmHg [18,19]. Concomitantly to oxygen levels change, there is a shift in the metabolism of neonatal CMs [20,21,22,23,24]. These significant changes in oxygen supply induce CMs maturation, terminal cell cycle withdrawal and polyploidization [24,25,26]. Considering that the mammalian heart has a limited capacity for regeneration compared to zebrafish, it has been hypothesized that low oxygen levels in CMs are essential for proper cardiac regeneration.

**Figure 1:**
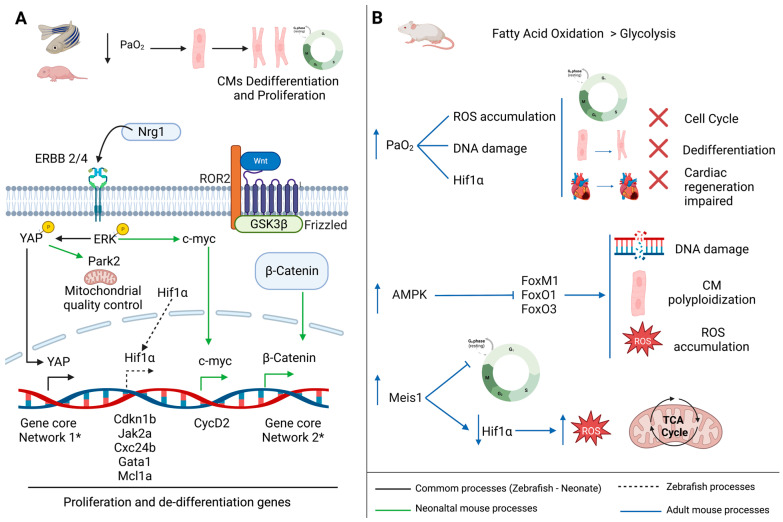
Comparison of the molecular pathways involved in cardiac regeneration mediated by hypoxia and oxidative metabolism among vertebrate species; (**A**) adult zebrafish and neonatal mouse and (**B**) adult mouse. * Different genes are involved.

Chronic low levels of O_2_ are needed for CMs proliferation and heart regeneration in zebrafish [27,28]. Concretely, in vitro experiments evidenced that a hypoxic environment promotes an increased amount of dedifferentiated CMs in zebrafish, promoting cell division [27] (Figure 1A). Jopling et al. (2012) identified a set of differentially expressed genes in Hif1α mutant amputated zebrafish hearts. They observed that during heart regeneration, a number of genes were upregulated in wild type samples during heart regeneration (Figure 1A). For example, those related with heart function, proliferation and dedifferentiation processes such as Thrombospondin 4b (thbs4b), Janus kinase 2a (jak2a), Thioredoxin interacting protein a (txnipa), Pim-1 proto-oncogen (pim1), Cyclin dependent kinase inhibitor 1b (cdkn1b), Solute carrier family 4-member 1a (slc4a1a), Chemokine (C-X-C motif) receptor 4b (cxcr4b), T-cell acute lymphocytic leukemia 1 (tal1), MCL1 Apoptosis regulator (mcl1a), Transforming coiled-coil containing protein 3 (tacc3) and GATA binding protein 1a (gata1a) [29,30,31,32,33,34,35,36]. It is worth mentioning that jak2a, mcl1a, cdkn1b, cxcr4b and gata1a are directly or indirectly regulated by Hif1α [27] (Figure 1A).

Some in vitro experiments demonstrated that cell cycle is enhanced in mammalian cardiomyocytes exposed to a hypoxic environment promoting CMs proliferative capacity [37,38]. Moreover, a few years ago, Nakada and coworkers, induced a chronic severe hypoxaemia in adult mice [16]. The gradual oxygen reduction leads to a decrease in ROS accumulation, reduced oxidative DNA damage and, moreover, to the stabilization Hif1α in CMs. These effects were enough to promote adult CMs cell cycle re-entry, improving cardiac functional recovering after MI [16]. A recent study with human heart samples and human induced pluripotent stem cell-cardiomyocytes (iPSC-CMs) corroborates that a moderate decreased O_2_ level promotes the expression of cell cycle markers [39].

### 3.2. Metabolic Switch Promotes Cardiomyocytes Cell Cycle Exit

As mentioned previously, increased oxygen supply after birth induces a metabolic rearrangement in mammalian CMs; during embryonic stages in CMs, ATP needs are met by glycolysis, whereas after birth, there is higher CMs energy demand, which is supplied through fatty acid oxidation, being closely related to mitochondrial expansion and the increase in reactive oxygen species (ROS) which exert oxidative DNA damage in CMs [20,21,22,23,24]. Several genes have been identified playing a key role in the switch from glycolysis to mitochondrial respiration, being implicated into the shift from proliferative to non-proliferative CMs [21,22,23]. For example, prenatally, Forkhead family transcription factors are the main regulators of CMs proliferation, namely, forkhead box (FOX) transcription factor M1 (FOXM1), FoxO1 and FoxO3 [40,41,42,43]. Sengupta et al. (2013) [44] identified that Fox transcription factors activity is regulated by the metabolic indicator AMP-activated protein kinase (AMPK), the activation of which is increased in mouse heart one week after birth. This lab also evidenced that AMPK inhibition in cultured neonatal rat CMs promotes cell-cycle activation [44] (Figure 1B). Moreover, myeloid ectopic viral integration site 1 homolog (Meis1) is increased after birth. This transcription factor promotes cell-cycle arrest in postnatal CMs [45]. Thus, it has been hypothesized that Meis1-Hif1α axis regulates cell metabolism as well as cell-cycle in CMs, as happens in hematopoietic stem cells [46] (Figure 1B). 

There are other important signaling pathways related with CMs metabolism and proliferation, i.e., Hippo [47,48,49,50,51,52,53,54,55,56,57], Wnt-B-Catenin [58,59,60] and Erk-Myc signaling pathways [58,59,61]. The Hippo-Yap signaling pathway, a kinase cascade that prevents adult CMs proliferation and regeneration, is upregulated in human HF [62]. Within the Hippo signaling pathway, the transcription factor Yap regulates CMs proliferation at embryonic stages. Moreover, it has been demonstrated that Yap activation in postnatal CMs stimulates cell proliferation in vitro and in vivo [48]. Moreover, the transcription factor Yap is phosphorylated by the extracellular signal-regulated kinase (Erk), mediated by the interaction of neuregulin 1 (Nrg1) with its receptor Erbb2 or Erbb4. This crucial step promotes Yap translocation to the nucleus, which is essential for CMs proliferation and heart regeneration in zebrafish and neonatal mice [53,54,55,56] (Figure 1A). Recent evidence has demonstrated that Erbb2 activation promotes CMs dedifferentiation and proliferation, triggering adult murine heart regeneration [55,56]. A few years ago, James Martin’s lab evidenced that the deletion of Salvador (Salv), another component of Hippo signaling, promotes the reparative genetic program in mouse hearts with ischemic HF [49]. Concomitantly, Parkin RBR E3 ubiquitin protein ligase (Park2), a Yap target gene related with mitochondrial quality control, is upregulated after Salv deletion, which is required for cardiac regeneration at P1 [49] (Figure 1A). Moreover, they evidenced that Paired-like homeodomain 2 (Pitx2) gain-of-function leads to adult mouse heart regeneration after apex resection. Concretely, Pitx2 participates in the activation of some components of the electron transport chain, as well as in the regulation of several genes related to ROS scavengers, regulating the antioxidant response, which is essential for cardiac repair [50].

The WNT/β-catenin signaling pathway promotes glycolysis through the stimulation of Wingless/integrated (WNT) target genes [63,64,65]. Related to this effect, Porrello’s lab evidenced that the WNT/β-catenin signaling pathway owns a dual role depending on the CMs stage. On the one hand, they observed that WNT/β-catenin leads to in vivo cell proliferation of neonatal mice CMs as well as in immature CMs-hiPSC, in vitro, through the induction of a core network of cell cycle-related genes [60] (Figure 1A). On the other hand, in the same study, they demonstrated that β-catenin has a cardioprotective role in adult CMs, ameliorating scar size post-MI [60]. 

Finally, Murray et al. (2015) demonstrated that redox activation of the ERK-cMyc signaling pathway promotes CMs proliferation through the increment of cyclin D2 [61] (Figure 1A). They developed a transgenic mouse model where the H_2_O_2_-generating enzyme NADPH oxidase 4 (Nox4) is overexpressed in postnatal CMs. In this scenario, Nox4 mediates the activation of the Erk1/2-cMyc signaling pathway, which results in the activation of cyclin D2 in 1–3-weeks-old mice, suggesting that this pathway could delay CMs exit from the cell cycle after birth [61]. 

Several studies regarding hypoxia and cardiac regeneration have evidenced that a low oxygenated environment is critical for the maintenance and regulation of CMs proliferation. In the same scenario the oxygen-dependent mitochondrial metabolism and ROS production are important in this regulation as well. These results suggest that modulating oxygen levels and myocardial metabolism could be a novel strategy to induce the new CMs formation.

## 4. Inflammatory Process in Cardiac Regeneration

The current hypothesis regarding the evolutionary drivers of the loss of cardiac regenerative potential in adult mammals has been proposed in recent years, such as the above-mentioned oxygen environment or endothermy acquisition. Thus, the transition to an oxygen-rich postnatal environment has been suggested to reduce CMs proliferative potential, inducing CMs cell cycle arrest and cardiac regenerative potential loss [14,66,67]. Vertebrate thermogenic capability, from ectothermy to endothermy, and increasing CMs ploidy appear to be inversely correlated with the cardiac regenerative potential during vertebrate evolution [68,69]. Comparative studies between species such as zebrafish, medaka, mice and humans have suggested that the development of robust inflammatory responses and a complex adaptive immune system parallels the decline of tissue regenerative potential [70]. According to this hypothesis, the capacity for regeneration and recovery after MI relies on the type and extent of immune response to cardiac injury. Thus, it has been proposed that mature and complex adaptive immune response in adult mammals compared to neonates and other evolutionarily ancient animals might be responsible for their limited regenerative capacity [71,72]. Moreover, the immune system appears to be a major difference between regenerative and non-regenerative models [72,73,74]. In fish, the importance of the early immune response during cardiac regeneration has been described as the reason for medaka’s inability to regenerate after ventricular resection [74]. In adult salamanders, primitive, less specific and slower-onset immune response has been related to a higher cardiac regenerative potential when compared with that observed in less-regenerative frogs [70]. In mammals, developmental maturation of the immune system is accompanied by the loss of scarless fetal regeneration [75]. This evidence suggests that the inflammatory response to cardiac injury is a critical regulator of the regenerative process, being able to drive tissue restoration, but also to inhibit it. 

**Figure 2:**
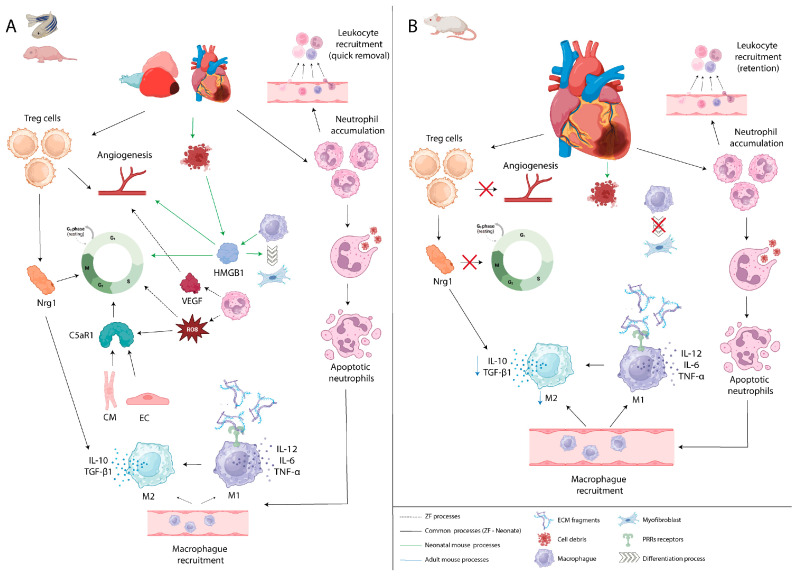
Comparison of the molecular pathways involved in cardiac regeneration mediated by inflammatory process among vertebrate species; (**A**) adult zebrafish and neonatal mouse and (**B**) adult mouse.

After cardiac damage, immune system activation in response to extensive cell death can be temporally divided into pro-inflammatory and inflammatory resolution/reparative phases, which play major roles in repair and regeneration. During the inflammatory period, myocardial fibers become necrotic and release endogenous molecules known as damage-associated molecular patterns (DAMPs), which foster the inflammatory response. For instance, high-mobility group B1 (HMGB1) is an MI-induced DAMP released by necrotic cells and/or secreted by macrophages that promotes, by interacting with pattern recognition receptors (PRRs), the maturation and migration of immune cells in a mouse ischemia-reperfusion (I/R) injury model [76,77,78]. HMGB1 promotes the activation and proliferation of stem cells, neoangiogenesis and the switch of cardiac macrophages into myofibroblasts [76,79] (Figure 2A). The activation of PRRs, present in immune cells, as well as in CMs or fibroblasts also propagates the inflammatory response through the induction and secretion of inflammatory cytokines and chemokines, including interleukin-6 (IL-6), IL-12 and Tumor Necrosis Factor alpha (TNF-α) [76,80,81,82,83,84,85] (Figure 2A,B). Thus, these endogenous molecules rapidly alert the immune system to tissue damage, mobilizing inflammatory leukocytes such as neutrophils and monocytes/macrophages by binding to PRRs, including Toll-like receptors (TLRs) [81,82]. For instance, dendritic cells respond to DAMPs through TLR2 and TLR4 and produce cytokines, such as IL-12, IL-13, and TNF, which are potent stimulators of the immune response [86,87]. In rodents, after MI, these cells have been reported to be accumulated early after injury in the damaged area, where they can activate FOXP3-CD4^+^ T helper cells and FOXP3^+^CD4^+^ regulatory T cells to prevent tissue-destructive autoimmunity [88,89].

Neutrophils, which dominate the damaged area in the first two days, are the most abundant incoming leukocytes after cardiac injury [90]. They release high levels of ROS, secrete proteases and pro-inflammatory mediators, exacerbate local injury and recruit other inflammatory leukocytes (Figure 2A). Despite being essential for initiating the acute inflammatory response, neutrophil activation needs to be tightly regulated to protect the host from excessive damage [91]. Moreover, neutrophils promote angiogenesis by secreting vascular endothelial growth factor (VEGF), and their arrival at the injured area precedes revascularization during heart regeneration in zebrafish [92] (Figure 2A). In zebrafish, early coronary invasion of the injured area has been described as a critical step to support the regenerative response; however, this response has not been observed in non-regenerative models such as medaka or mice [93]. Although both regenerative and non-regenerative systems display swift neutrophil deployment in injured hearts, differences in the timing of neutrophil retention/resolution and cell subtypes likely contribute to repair or regeneration outcomes. Nevertheless, excessive and prolonged neutrophil activity is largely associated with an unresolved inflammatory response and potentially affects cardiac repair and regeneration [94]. Quick neutrophil recruitment and resolution likely contribute to regenerative mechanisms, whereas lengthy neutrophil retention and elevated neutrophil numbers in non-regenerative systems hinder the repair process (Figure 2A,B). However, the precise spatial and temporal regulation of the inflammatory response toward scar-free regeneration after cardiac injury, such as MI, remains unclear [95]. Comparative analyses of the cardiac regenerative capacity between zebrafish and medaka revealed that delayed recruitment of macrophages disrupts neovascularization and neutrophil clearance after cardiac injury in a non-regenerative model [74]. Similarly, in adult mice, insufficient neutrophil removal leads to enhanced matrix degradation and increased susceptibility to cardiac rupture [96].

Upon engulfment of cell debris, neutrophils undergo apoptosis and activate signals that promote their clearance by macrophages [97]. These “eat me” indications polarize macrophages towards a reparative phenotype, inducing inflammation resolution by the expression of anti-inflammatory and reparative cytokines, such as IL-10 or transforming growth factor-β1 (TGF-β1), and contributing to the resolution of the inflammatory phase [98,99] (Figure 2A,B). Another critical component of innate immunity, the complement receptor gene *C5aR1,* is activated in CMs and endothelial cells (ECs) after cardiac injury in regenerative models, such as axolotl, zebrafish and neonatal mice, and its inhibition significantly attenuates CMs proliferation [100] (Figure 2A). ROS released from the mitochondria of necrotic cells or secreted by neutrophils can also activate the complement system, promoting immune cell infiltration and direct activation of the inflammasome in cardiac fibroblasts (CFs) and mast cells (Figure 2A). Inflammasome activation leads to the maturation and secretion of pro-inflammatory cytokines by CFs such as IL-1β and IL-18 [101,102]. Although this inflammatory response is essential for clearing necrotic cells and activating CMs dedifferentiation and proliferation, excessive ROS generation extends myocardial injury, causing DNA damage and cell cycle arrest in CMs [103].

As mentioned above, immune cells recruited to the injured tissue clear debris, dead cells and degrade the extracellular matrix (ECM) [104]. The resulting ECM fragments contribute to inflammatory propagation by regulating leukocyte engagement via integrin receptors and stimulating macrophage chemokine secretion via TLRs [105,106,107] (Figure 2A,B). These transmembrane receptors are important mediators of post-infarction inflammatory reactions and their signaling seems to trigger CMs proliferation during regeneration [82,108,109]. For instance, administration of TLRs agonists preconditions CMs for cell cycle re-entry in zebrafish, induces CMs proliferation in the neonatal mouse heart and in non-regenerative medaka, promotes macrophage recruitment, revascularization and CMs proliferation [74,110,111]. The ECM also provides signal transduction, which serves as a scaffold for migrating inflammatory cells and supports the proliferation of ECs and fibroblasts during cardiac repair [112]. Interestingly, ECM synthesis genes were among the most upregulated post-injury genes in both neonatal mouse and zebrafish hearts, suggesting that the main role of ECM after cardiac injury is a common characteristic in both regenerative and non-regenerative models [100,112,113].

Similarly, DAMP-PRRs activation on fibroblasts, the second largest population of cardiac cells, also plays a crucial role during the pro-inflammatory phase, altering the ECM turnover, the production of fibrotic and inflammatory paracrine factors and promoting its transdifferentiation into myofibroblasts. Fibroblasts are indispensable players in cardiac repair, which is dependent on the formation of a collagen-based scar to maintain structural integrity. In regenerative models such as zebrafish, fibroblasts contribute to heart regeneration by promoting scar resolution during post-infarct healing [114,115]. 

As mentioned before, damage signals trigger inflammation in both resident cells (fibroblasts, ECs, CMs, epicardial cells, etc.) and recruit immune cells (neutrophils, lymphocytes, etc.), such that the outcome following cardiac injury appears to depend largely on the number, kinetics and phenotypes of these cells. Lymphoid cells, such as Tregs, have been described as critical players in myocardial healing by promoting revascularization and macrophage differentiation toward the M2 phenotype; however, in adult mammals, this adaptive immune system might be responsible for their limited regenerative capacity [71,116,117]. In zebrafish and mice, Tregs have been reported to promote CMs proliferation in neonatal hearts and after MI in adults through the secretion of the CMs mitogen Nrg1 [118,119] (Figure 2A,B). The adaptive immune response, mediated by lymphocytes, has been described as an important difference between regenerative animals, such as fish and salamanders, with sophisticated innate immune strategies that reduce the dependency on adaptive immunity, and those animals with a limited regenerative capacity that have a highly specialized adaptive immune response against cardiac injury [120]. Thus, whereas T-cell quick removal supports inflammation resolution, promoting new contractile tissue formation and coronary revascularization in regenerative models, extensive T-cell persistence contributes to a lengthy inflammatory response in non-regenerative systems, altering cardiac remodeling and inducing heart failure development [121].

The transition to a pro-reparative phase several days after cardiac injury is marked by the repression of pro-inflammatory signals and, although the signals that induce the termination of the inflammatory phase are not fully understood, the crosstalk between monocytes, macrophages and fibroblasts is likely to play a major role containing the inflammatory response [122,123,124]. Lymphocyte subpopulations are key effectors implicated in the negative regulation of inflammation as they are recruited to the damaged area where they express anti-inflammatory cytokines such as IL-10, promote differentiation of macrophages to an anti-inflammatory phenotype and modulate protease synthesis by CFs [116,125,126]. Moreover, during this period, monocytes, macrophages and differentiated ECs coordinate angiogenesis to form new blood vessels, providing blood supply to the damaged area [94]. In this region, the proliferation and differentiation of fibroblasts into myofibroblasts and collagen deposition generate the granulation tissue. In non-regenerative adult mammals, the maturation phase of tissue repair forms a non-contractile scar at the injury site, contributing to HF development [127].

Recent studies on heart regeneration in different experimental models have revealed that the immune response after cardiac injury is a differential feature between animals that can regenerate their hearts and non-regenerative models. Although the mechanisms of heart regeneration among model organisms appear remarkably similar, starting with an inflammatory phase, the following period, known as the reparative phase, differs between animal groups and can contribute to the development of HF. Modulation of immune cellular processes, including macrophage polarization and T-cell activation into Tregs, may represent a promising direction to promote cardiac healing and even regeneration. 

## 5. Reactivation of Cell Proliferation for Heart Repair

**Figure 3:**
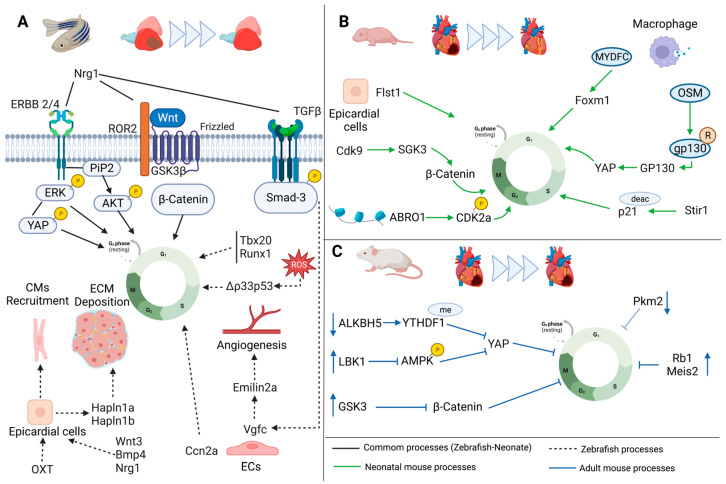
Comparison of the molecular pathways involved in cardiac regeneration mediated by proliferation process among vertebrate species; (**A**) adult zebrafish, (**B**) neonatal mouse and (**C**) adult mouse.

After MI, adult mammalian CMs are not able to re-enter into the cell cycle and this effect leads to the loss of billions of CMs, which are replaced by fibrotic myocardial tissue that results in the loss of cardiac function and eventually HF. The scientific research community is focused on studying the possibility of making CMs more proliferative and consequently able to recover cardiac function [128] (Figure 3A–C). 

### 5.1. The Myocardial Inductors of Cardiomyocyte Proliferation

#### 5.1.1. Cell Signaling

Several signaling pathways are key mediators of CMs proliferation, such as Hippo-YAP [129,130], neuregulin [131,132] and fibroblasts growth factor [133]. Within the Hippo-YAP signaling pathway [129,130], Cai’s lab evidenced that the α-ketoglutarate-dependent dioxygenase alkB homolog 5 (ALKBH5), a N^6^-methyladenosine (m^6^A) eraser, is decreased in the heart after birth. ALKBH5 knockout decreases heart regeneration capacity after apex resection in mice, whereas its overexpression enhances regeneration in neonatal P7 and adult mice after MI [134]. Concretely, ALKBH5 promotes CMs proliferation through the stabilization of the YTH N^6^-methyladenosine RNA-binding protein 1 (YTHDF1) by demethylation, increasing its expression and promoting YAP translation, inducing CMs to re-enter the cell cycle [134] (Figure 3C). During pregnancy, circulatory hormone levels are crucial for proper fetal development and growth [135]. Progesterone levels decline after birth, being concomitant with the loss of heart regeneration capacity. Recent pieces of evidence demonstrated that progesterone supplementation leads to progesterone receptor interaction with YAP promoter, upregulating YAP target genes. This molecular regulation promotes CMs proliferation and improves cardiac function in an adult mice MI model [135]. Oncostatin M (OSM) is one of the upregulated cytokines during neonatal heart regeneration in mice. OSM is secreted by macrophages during regeneration, promoting CMs proliferation through OSM receptor heterodimers OSMR/gp130 (glycoprotein 130) [136] (Figure 3B). Particularly, OSM binds to OSMR/gp130 heterometric receptor and activates gp130, inducing CMs de-differentiation and proliferation through the activation of the Src-Yap signaling pathway, promoting myocardial regeneration and cardiac repair in neonatal mice [136]. Finally, Qu et al. [137] observed that liver kinase B1 (LKB1) levels are rapidly increased after birth (Figure 3C). Through loss- and gain-of-function experiments in rats, they demonstrated that LKB1 levels negatively correlated with CMs proliferation [137]. LKB1 in vitro knockdown significantly represses AMPK phosphorylation, leading to YAP activation and, thus, the promotion of CMs proliferation. This study sets LKB1 as a possible therapeutic target for stimulating CMs proliferation [137]. Another important effector of CMs proliferation is Nrg1, as mentioned above, and its tyrosine kinase receptors, ERBB2 and ERBB4 [55,56,138]. Several laboratories have evidenced that ERBB2 expression levels decrease after birth, while ERBB2 overexpression promotes CMs proliferation after an injury [55,56]. Moreover, YAP, ERK, AKT and GSK3β/β-catenin signaling pathways are activated downstream of ERBB2 signaling, promoting CMs proliferation and EMT-like processes during cardiac regeneration in mice [55,56,138] (Figure 1A). In an MI rat model, the administration of microparticles loaded with Fibroblast growth factor 1 (FGF1) and Nrg1 after MI leads to cardiac function improvement, smaller infarct size, reduction of fibrosis and induction of tissue revascularization [139]. Similarly, FGF1 administrated with a p38 inhibitor in a rat MI model improves heart regeneration through the enhancement of angiogenesis and CMs mitosis [140]. Regarding the role of Nrg1 in the regulation of CMs proliferation, Shoffner et al. (2020) observed that the Tumor suppressor p53 (Tp53) protein’s levels fluctuate during heart regeneration in zebrafish, while the levels of its negative regulator, an E3 ubiquitin ligase Mouse double munite 2 homolog (Mdm2), are increased [141]. They observed that the Tp53 network is transiently suppressed during zebrafish heart regeneration, increasing CMs proliferation, indicating that this molecular pathway is directly related to innate regeneration in zebrafish [141]. In addition, zebrafish ex vivo experiments performed by Arora et al. [142] demonstrated that the combination of Wnt family member 3 (Wnt3), Bone morphogenetic protein 4 (BMP4) and Nrg1 has a regenerative effect in damaged neonatal hearts in culture. The combination of these growth factors promotes cardiac repair via epicardial cell activation, their proliferation and migration to the injury site, followed by CMs recruitment [142] (Figure 3A). Finally, Nrg1 regulates CMs proliferation through the activation of the Ras/Mapk pathway, which is controlled by feedback attenuator Dual specificity phosphatase 6 (Dusp6), an ERK phosphatase [143] (Figure 3A). Tsang’s lab evidenced that in rat primary CMs in vitro, the chemical inhibition of Dusp6 stimulates Nrg1, enhancing CMs proliferation. Moreover, this effect is recapitulated in a zebrafish resection model, where the suppression of Dusp6 enhances cardiac repair through the promotion of CMs proliferation, coronary angiogenesis and reduced fibrosis. [143]. 

Furthermore, it is well known that the other signaling pathway which is involved in cell proliferation is PI3k/AKT/GSK3B. Glyocogen synthase kinase 3 (Gsk-3) is a serine/threonine Kinase with two isoforms, GSK3A and GSK3B. GSK-3 mediates phosphorylation of β-catenin, leading to its ubiquitination and degradation, decreasing the free β-catenin to export into the nucleus, where induces YAP expression [144]. GSK3B knockout promotes CMs proliferation after MI in mice. Mechanistically, GSK3B suppresses the expression and secretion of growth factors required for CMs proliferation [145] (Figure 1A). Within this scenario, Small ubiquitin-like modifier-specific protease 2 (SENP2) deficiency, in mice, promotes P7 and adult CMs de-differentiation and proliferation in vitro and in vivo. The loss of SENP2 upregulates AKT deSUMOylation, increasing its activity, leading to a decrease of GSK3B levels and promoting CMs proliferation and angiogenesis [146]. In addition, the Cyclin-dependent kinases (CDK) family is closely related to cell proliferation. CDK9 expression, the most representative member of the CDK family, declines its levels in the myocardium from newborn to adulthood [147] (Figure 3B). CDK9 is located upstream of the threonine-protein kinase 3 (SGK3), a functional kinase with the capacity to promote CMs proliferation and cardiac repair after MI. Gain- and loss-of-function studies show that CDK9 promotes cardiac regeneration after apical resection in neonatal mice and its overexpression promotes mature CMs to re-enter the cell cycle, activating SGK3 and the downstream GSK3B/β-catenin pathway [147]. Moreover, SGK3 promotes β-catenin expression and also upregulates cell cycle genes like Cyclin-D1, c-myc and Cdc20, and downregulates the expression of cell cycle negative regulators such as cyclin kinase inhibitor p21 and cyclin kinase inhibitor p27 [148] (Figure 3B). Finally, few years ago, Magadum et al. (2020) demonstrated that Pyruvate kinase muscle isozyme (Pkm2) is an important regulator of the CMs cell cycle. Pkm2 is expressed during development, but just after birth, its expression levels decrease [149] (Figure 3C). Loss-of-function studies during mouse development show that Pkm2 reduces CMs number and myocardial size. However, using Pkm2-modified RNA after MI leads to an increased rate of CMs division, enhanced cardiac function and reduced oxidative stress damage through β-catenin and anabolic pathways [149].

#### 5.1.2. Transcription Factors

**Figure 4:**
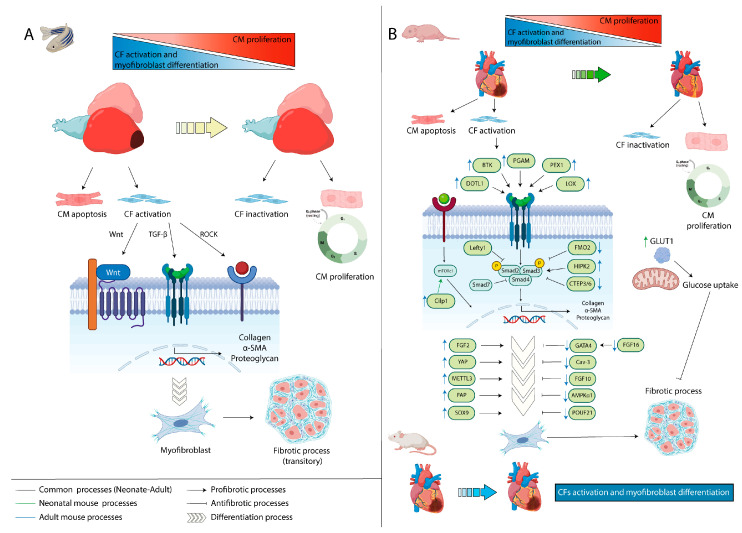
Comparison of the molecular pathways involved in cardiac regeneration mediated by fibrotic process among vertebrate species; (**A**) adult zebrafish, (**B**) neonatal and adult mouse.

There are other molecular effectors which also control the CMs cell cycle. For example, Retinoblastoma 1 (Rb1) and Meis homeobox 2 (Meis2) are cell cycle inhibitors [150,151] (Figure 3C). It has been further evidenced that their suppression promotes adult CMs re-entry to the cell cycle. Concretely, Alam et al. (2019) [150] observed that the simultaneous inhibition of both, in adult rat CMs and in human iPSc-CMs, results in an increase in the cell number and the mononucleated CMs in vitro. Moreover, Rb1 and Meis2 silencing after MI improves cell survival and increases proliferation by reducing infarct scar size and improving cardiac function [150,151]. Furthermore, last year, it was elucidated the dual role of FGF10 [152]. To be more precise, FGF10 enhances heart repair by promoting CMs renewal and reducing fibrosis. These effects are observed when FGF10 levels are upregulated in the injured ventricle after MI in adult mice, activating major regenerative pathways including the regulation of Meis1 and the Hippo signaling pathway [152] (Figure 4B). On the other hand, GATA4 is a cardiac transcription factor required for neonatal mouse heart regeneration. In an inducible model of GATA4 knockout, it showed reduced CMs replication, impaired coronary angiogenesis and increased hypertrophy and fibrosis after injury. In addition, FGF16 is downregulated in GATA4 knockout, whereas overexpression of FGF16 rescues heart regeneration, promoting CMs replication and improvement of heart function after injury [153].

### 5.2. The Epicardial Inductors of Cardiomyocyte Proliferation

#### 5.2.1. Cell Signaling

Moreover, it needs to be considered that heart regeneration is not only driven by the CMs proliferation in the myocardium, the epicardium also plays an important role during cardiac repair, i.e., secreting factors that are essential for CMs proliferation [154]. For example, follistatin-like 1 (Fstl1) released from the epicardium activates CMs proliferation after MI. The ability of Fstl1 to promote CMs proliferation depends on its post-translational modification to a glycosylated state. Thus, Magadum et al., 2018 evidenced that the administration of Fstl1 modRNA with the N180Q mutation on its N-glycosilation site directly in the infarcted myocardium can mimic the regenerative effect of regular Fstl1 secreted from the epicardium, leading to CMs proliferation and cardiac regeneration in mice [155]. Moreover, recent findings from Neef’s lab report that proliferation of matured hypoxic iPSC-CMs is promoted by the Fstl1 secreted by CFs [156] (Figure 3B). In zebrafish, a particular cluster of epicardial cells has a strong association with regeneration and is marked by the expression of Hyaluronan And Proteoglycan Link Protein 1 (Hapln1a) and Hapln1b. The depletion of Hapln1 expression in cells, or its genetic inactivation, alters key ECM deposition, disrupting CMs proliferation and inhibiting heart regeneration [157] (Figure 3A). Similarly, oxytocin (OXT) signaling is critical for proper epicardium development in zebrafish. After cardiac cryoinjury in zebrafish, OXT is produced, leading to epicardial activation, which promotes heart regeneration [158] (Figure 3A). Moreover, after cardiac injury in zebrafish, ECs upregulate Vascular endothelial growth factor c (Vegfc). Blockage of Vegfc signaling reduces CMs dedifferentiation and proliferation. Moreover, Elastin Microfibril Interfacer 2 (Emilin2a) is a target of Vegfc signaling and its expression can modulate coronary revascularization as well as CMs proliferation [159] (Figure 3A). The TGF-β/Smad3 signaling pathway has also been implicated in cardiac regeneration. For instance, the inhibition of Smad3 in a model of ventricular ablation in zebrafish reduces CMs proliferation and migration; concretely, the cell cycle is disrupted and EMT response is impaired, limiting cardiac regeneration [160] (Figure 3A). Mukherjee et al. (2020) observed in zebrafish that Cellular communication network factor 2a (Ccn2a) is induced in ECs in the injured tissue and regulates CMs proliferation (Figure 3A). The mechanism involves the enhanced expression of pro-regenerative ECM genes through the modulation of the TGF-β/Smad3 signaling pathway [161]. AZD6244 is a MEK inhibitor that induces a reduction of pERK in a ventricle resection model in zebrafish, and its administration after injury decreases CMs proliferation. Moreover, pERK is also expressed in non-CMs nearby the injured area, as in the epicardium and endocardium during regeneration, suggesting that the role of the MAPK/ERK signaling pathway is related to cardiac remodeling by influencing angiogenesis or ECs migration [162]. A downstream effector of MEK is the mitogen-activated protein kinase (Mapk)-interacting serine/threonine-protein kinase 2 (MNK2), which is elevated at the infarct border zone in mice. MNK2 binds to Eukaryotic Translation Initiation Factor 4E (EIF4E), regulating its phosphorylation, which activates cyclinD1 (CycD1), promoting CMs proliferation and leading to cardiac repair [163]. 

#### 5.2.2. Transcription Factors

Additionally, the modulation of several transcription factors has been proposed as regenerative promoters. For example, Fang et al. (2020) identified that T-Box transcription factor 20 (Tbx20) is induced rapidly in myocardial wound edge and in the atrial epicardium in zebrafish. Such Tbx20 induction also plays a role in ECs migration and regeneration through upregulation of endocardial BMP6 signaling after damage. In addition, overexpression of Tbx20, specifically in adult CMs after injury, promotes CMs dedifferentiation and proliferation [164] (Figure 3A). Another transcription factor highly expressed in several cell populations of the zebrafish heart after injury is the Runt-Related Transcription Factor 1 (Runx1). Mommersteeg’s lab reported that the complete absence of Runx1 in zebrafish mutants promotes myocardial survival and proliferation and impairs fibrosis through the upregulation of several components of the fibrin degradation pathway, leading to reduced collagen and fibrin deposition in mutant wounds (Figure 3A). This study highlights that, although zebrafish have the potential of heart regeneration, it may not be optimal, a fine balance being needed among fibrosis and CMs proliferation [165]. As mentioned previously, the transcription factor Foxm1 is overexpressed in the border zone after heart injury in zebrafish, promoting CMs proliferation. It has been observed that Foxm1 mutants decrease CMs proliferation and the expression of genes associated with cell cycle checkpoints. Moreover, low levels of Centromere Protein F (CENPF), a Foxm1 target, which is a microtubule and kinetochore binding protein, regulates CMs binucleation, impairing mitosis [166]. In addition to Foxm1 regulation, Wang et al. (2020) identified Myeloid-derive growth factor (MYDFG), a paracrine protein secreted by monocytes and macrophages, as a protective gene against cardiac injury in adult mice. Concretely, in neonates, its expression is predominantly observed at ECs. Thus, MYDFG deficiency impairs neonatal heart regeneration and CMs proliferation. MYDGF regulation is mediated by the activation of the c-Myc/Foxm1 pathway and improves regeneration in neonate and adult mice after cardiac injury [167] (Figure 3B).

### 5.3. Other Mediators of Cardiomyocyte Proliferation

The study of the chromatin landscape and epigenetic barriers in a healing heart could be useful to identify transcription factor regulators that promote CMs regeneration. Abraxas 2 (ABRO1), a component of the deubiquitinating system, regulates CMs and cardiac regeneration by targeting the hypermethylation of Phosphoserine phosphatase (Psph) which dephosphorylates CDK2, a positive regulator of the cell cycle [168,169]. Moreover, knockout mice of the cyclin-dependent kinase inhibitor 2A (Cdk2a) show enhanced CMs proliferation in vitro and in vivo (Figure 3B). Heart function was improved and the scar size decreased in Cdk2a knockout mice after ischemia reperfusion injury [170]. In the same line, activator protein-1 (AP-1) binds to DNA regions, promoting a gain of accessibility during cardiac regeneration. Blocking AP-1 function in zebrafish leads to defects in CMs proliferation as well as decreased chromatin accessibility of F-Box and Leucine Rich Repeat Protein 22 (Fbxl22) and Integrin Linked Kinase (Ilk) factors that regulate sarcomere disassembly, a process required for the dedifferentiation and proliferation of CMs [171]. On the other hand, it has been evidenced that deacetylation of histone tails by histone deacetylases (HDACs) promotes chromatin condensation and the repression of gene expression. Particularly, the loss of Hdac1 in zebrafish leads to a reduction of CMs proliferation after injury [172]. Finally, previous studies indicate that acetylated p21 induces cell cycle arrest, inhibiting CMs proliferation and cardiac regeneration. Sirt1 deacetylase reduces p21 levels, increasing CMs proliferation in neonatal and adult mice post-MI [173] (Figure 3B). P53 signaling is activated during cardiac regeneration and triggered by ROS. Concretely, p53 isoform Δ133p53 is highly expressed upon stimulation by low-level ROS, and this isoform in coordination with p53 promotes cell survival by mediating the expression of antioxidant genes. In a zebrafish heart resection model, the expression of Δ133p53 and p53 is activated, promoting CMs proliferation and, thus, contributing to myocardial regeneration [174] (Figure 3A).

Furthermore, as mentioned previously, pro- and anti-inflammatory steps are crucial for cardiac regeneration. In this regard, several labs have performed some studies and have evidenced that, for example, the global deletion of IL13Rα1 decreases CMs proliferation during early postnatal development and impairs cardiac regeneration in neonatal mice [175]. Moreover, IL-4 and IL-6, secreted by macrophages M2 during cardiac regeneration, induce ECs proliferation and enhance CMs proliferation after injury [176,177].

Finally, transplant experiments in mice by using isolated cardiac progenitors’ cells derived from mouse embryonic stem cells (mESC), treated with the hormone and cardiokine Fibronectin Type III Domain-Containing 5 (Irisin), promote CMs proliferation in the injured area, attenuating myocardial fibrosis, promoting regeneration and neovascularization. Moreover, in vitro experiments evidenced that irisin treatment reduces HDAC4, while increasing p38 acetylation. These results point out irisin stem cell treatment as a novel therapeutic process for cardiac repair (ES LA 250 Zhao IRISIN) [178].

Overall, the scientific research community is making great efforts to elucidate the key for the improvement of CMs proliferation in adult mammals after injury. To do this, it is important to decipher the mechanisms that arrest CMs proliferation after birth and to discuss new possibilities for potential clinical treatment development to face cardiovascular diseases.

## 6. Modulation of Extracellular Matrix Deposition during Cardiac Repair

In response to a loss of CMs after a cardiac injury, fibrosis is one of the first processes that participates in cardiac remodeling. Frequently, cardiac fibrosis is necessary for the maintenance of the cardiac structure [179]. As mentioned above, due to the low regenerative capacity of mammalian CMs after cardiac damage, this cell population is replaced by CFs, being fibroblasts, in general terms, the most abundant cell type in connective tissue and participating in the structural support of several organs [180]. In the MI pathological context, some CFs are activated and differentiated into myofibroblasts, which have characteristics of both smooth muscle cells and fibroblasts and have high levels of alpha-smooth muscle actin (α-SMA) [181,182]. Another important element in the fibrosis process is the ECM, where one of its main components is collagen. Collagens help to maintain tissue integrity through its interaction with other ECM molecules, as well as with growth and differentiation factors [183,184,185]. Moreover, there are other elements in ECM, such as glycosaminoglycans, glycoprotein, growth factors or proteases, which are activated after cardiac damage, leading to a reparative response [179]. After a myocardial injury, dynamic changes in the composition of the ECM occur, contributing to the reparative cellular responses and leading to fibrosis development to prevent ventricle rupture [186,187,188]. In this review, we have described that the inflammatory process affects ECM integrity [96] and, moreover, the transcription factor Runx1 impairs fibrosis [165], indicating that fibrosis is a tightly regulated process, as described as follows, depending on the animal species.

### 6.1. Zebrafish

The fibrotic process occurs transiently in the zebrafish model since CFs are inactivated, gradually allowing CMs regeneration [114,189] (Figure 4A). However, if the fibrotic response is maintained over time, the tissue becomes more rigid, disrupting cardiac function [190,191], giving rise to the fibrotic process observed in mammals. Unlike mammals, zebrafish have low fibrosis because they have an almost complete cardiac remodeling capacity due to the reactivation of CMs proliferation [192,193]. However, mammalian CMs are replaced by CFs, and as the degree of CFs differentiation increases, the remodeling capacity decreases [182] (Figure 4B). In rodents, it has been shown that after MI, the remodeling capacity is higher in P1 than at later (P2–P7) and adult stages, when fibrosis is very significant [194,195], and thus, changes in metabolism are a possible cause of these differences in regenerative capacity. As mentioned previously, after birth, the heart changes its main substrate from glucose to fatty acids, coinciding with the loss of regeneration [196]. In this regard, it has been evidenced that the overexpression of the embryonic form of constitutively active glucose transporter (GLUT1) allowed a greater uptake of glucose and a reduction of fibrosis in neonatal mice after cryoinjury [197] (Figure 4B). Therefore, in mammals, irreversible fibrosis is produced, and although in neonatal stages there is some regenerative capacity, this is limited and very different from zebrafish [198].

To achieve cardiac remodeling, it is necessary to control the regulation of gene expression and post-translational modification of the main proteins implicated in ECM modulation, particularly collagens [199]. Several pathways participate in the regulation of fibrosis, some of them promoting fibrosis, for example, TGF-β, WNT signaling or the Rho-kinase pathway (ROCK). Furthermore, some growth factors and other signaling molecules (FGF2, FGF10, VEGFC) also could modify these signaling pathways, regulating the fibrosis process [181] (Figure 4A). Finally, as described in the previous paragraphs, other different activated processes after MI, such as proliferation and inflammation, could regulate fibrosis, since several molecules block cardiac remodeling, while others promote CMs regeneration [96,165]. This is the reason why many studies focus their attention on the molecular regulation of the main signaling pathways implicated in fibrosis to develop new strategies to reduce fibrosis after cardiac injury.

### 6.2. Mice

The fibrotic response in mice after cardiac injury is much more complex than in zebrafish. The TGF-β/Smad pathway, involved in most fibrotic processes, has two different routes: (1) Canonical pathway or Smad-dependent; (2) Non-canonical or Smad-independent. In the canonical route, the TGF-β2 receptor activates the TGF-β1 receptor, promoting the phosphorylation and activation of Smad2 and Smad3. Subsequently, Smad4 binds to the Smad2/Smad3 complex, regulating fibrotic gene transcription. Other Smad proteins, Smad6 and Smad7, have an inhibitory role. The non-canonical route is based on the regulation of the MAPK, Rho, PI3K, AKT or NF-Kβ pathways [200,201,202].

Within the canonical route, the expression of the Homeodomain-interacting-protein-kinase 2 (HIPK2) is increased in the myocardium from transverse aortic ligation (TAC) in mice, as well as in the left ventricle of HF patients. These elevated expression levels promote fibrosis through the activation of Smad3 after its phosphorylation (Figure 4B). Thus, the inhibition of HIPK2 could reduce the CFs proliferation and differentiation, improving the fibrotic process [203]. The phosphorylation of Smad2 and Smad3 is an important step in the TGF-β/Smad pathway, and it is regulated by Flavin-containing monooxygenase 2 (FMO2), whose expression levels are reduced in rats with MI (Figure 4B). FMO2 is implicated in the recruitment of cytochrome p450 superfamily 2J3 (CYP2J3), blocking the CYP2J3/SMURF2 interaction and promoting the nuclear translocation of Smad2 (switching to its inactive form). For this reason, FMO2 has an anti-fibrotic role after cardiac injury, being a possible therapeutic target [204]. Li et al. (2021) [205] also described a member of the TGF-β family, Left-right determination factor (Lefty1), with an anti-fibrotic function, since both in vitro and in vivo assays in mice show that Lefty1 reduces CFs proliferation and differentiation through the inhibition of phosphorylation of Smad2 and ERK1/2 after MI [205] (Figure 4B). Moreover, an anti-fibrotic role is also described in the MI rodent model (mice/rats) for several other molecules. C1q/tumor necrosis factor-related protein-3 (CTRP3) is an adipokine whose expression is reduced after MI and causes an increase in α-SMA and pro-fibrotic genes expression (Figure 4C). This adipokine promotes cardiac remodeling by the activation of AMPK/AKT signaling, which inhibits Smad3 and myofibroblast differentiation [206]. Other molecules with an anti-fibrotic role are FOXF1, GSK-3β (also in human) and SH2 domain-containing protein tyrosine phosphatase-2 (SHP-2), which also inhibit TGF-β/Smad3 activation [202,207,208]. According to the anti-fibrotic role of Smad7, it has been demonstrated that Smad7 is also activated after MI to regulate excessive fibrosis and can inhibit Smad2/Smad3, ERK and AKT pathways without affecting the TGF-β1 receptor. Furthermore, Smad7 is a direct target of ErbB2, inhibiting EGFR/ErBb2 signaling, reducing the fibrotic process [209] (Figure 4B). In addition to these findings, the regulation of the canonical route of the TGF-β/Smad pathway can be exerted by other molecules, i.e., phosphoglycerate mutase 1 (PGAM1) or Bruton’s tyrosine kinase (BTK), promoting a pro-fibrotic situation [210,211] (Figure 4B). In this context, high levels of PGAM1, observed in mice after MI by left anterior descending coronary artery (LAD) ligation, increase the TGF-β/Smad2/3 activation by directly activating TGF-β [210]. The increase in BTK expression due to MI also activates this signaling pathway by targeting TGB-β receptor I, raising the differentiation from CFs to myofibroblast and other ECM genes and, thus, increasing fibrosis [211]. Moreover, as mentioned in the regulation of proliferation process, epigenetic modification could also regulate cardiac fibrosis through the TGF-β/Smad pathway. For example, Li et al. (2022) [212] demonstrate that high levels of Disruptor of telomeric silencing 1-like (DOTL1) caused by LAD-induced MI produce high increments of Spleen tyrosine kinase (SYK) by increasing the H3K79me2 modification of the SYK promoter. This epigenetic modification is related to higher activation of the TGF-β/Smad3 pathway, promoting cardiac fibrosis [212] (Figure 4B). Finally, it is interesting to highlight that the pro-fibrotic effect that some proteins could be cell type-specific. An example is the transcription factor Phenylephrine-induced complex-1 (PEX1), which is upregulated after MI. However, this transcription factor only acts in ventricular fibroblasts, but not in ventricular myocytes. PEX1 upregulates metalloproteinase 9 (MMP9), which activates TGF-β signaling and myofibroblast differentiation [213] (Figure 4B). Due to TGF-β activation following cardiac injury, other proteins that can promote fibrosis are subsequently activated, such as Limb-bud and heart (LBH), which is a target of TGF-β. The activation of LBH allows its interaction with αβ-crystallin (CRYAB), producing an increase in CFs proliferation and myofibroblast differentiation [214]. Another protein is Lysil oxidase enzyme (LOX), which is upregulated by the TGF-β activation after cardiac injury in both in vitro and in vivo assays. This enzyme catalyzes collagen and elastin fibers cross-linking, promoting a fibrotic scar [215] (Figure 4B).

Within the non-canonical route, several studies show that the fibrotic process can also be regulated by other signaling pathways, i.e., MAPK signaling, which has already been described in the context of proliferation [143,162,216]. In a fibrotic situation, Dusp6 is reduced after ventricular resection in zebrafish, associated with an inhibition of MAPK signaling and the fibrotic process [143]. The relationship between fibrosis and MAPK inhibition is also studied by Tian et al. (2020) [216] through Anoctamin-1 (ANO1) protein. These authors observed that in a rat MI model by LAD, ANO-1 is upregulated, especially in CFs. RNA-seq analyses show that ANO-1 controls MAPK signaling by the regulation of Angiotensin II type I receptor (AT1R) and the phosphorylation of MEK and ERK1/2, which are central elements of MAPK signaling. These authors propose ANO-1 inhibition as a tool to reduce fibrosis after cardiac injury [216].

Another pathway implicated in fibrosis is mTORC1, which is regulated by Cartilage intermediate layer protein 1 (Cilp1), an ECM protein that promotes myofibroblast proliferation. Cilp1 is mainly expressed in CFs, and in mice with MI by LAD, and in human samples with MI, high levels of Cilp1 are detected. This upregulation produces more myofibroblast proliferation due to mTORC1 activation [217] (Figure 4B). The increase in myofibroblast differentiation is also regulated by C1q/tumor necrosis factor-related protein-6 (CTRP6), whose expression diminished in rat with MI by LAD. The downregulation of this protein activates TGF-β1 and myofibroblast differentiation, although the canonical route is not activated. TGF-β1 activation regulates the Rhoa/MRTF-A pathway, promoting cardiac fibrosis [218] (Figure 4B).

Several studies show that transcription factors or growth factors could exert an important function in fibrosis [219,220,221], such as the transcription factors Sex-determining region Y box 9 (SOX9) or POU-domain transcription factor (POU2F1), which have opposite functions in the fibrotic process. SOX9 is a pro-fibrotic factor that is upregulated in scar after MI in mice. Scharf et al. (2019) induced fibroblast-specific SOX9 deletion in mice, where an improvement of cardiac function was observed after MI. Moreover, SOX9 deletion reduces the scar area and inhibits the expression of several genes related to ECM and the pro-inflammatory process, thus controlling the CFs differentiation [219] (Figure 4B). On the other hand, POUF21 can bind to the promoter of anti-fibrotic genes when the tissue becomes permanently stiffened, inhibiting their expression in both in vitro and in mice with LAD-induced MI [220] (Figure 4B). 

Yu et al. (2016) [153] describe a regulatory network between transcription factors and growth factors. In a neonatal mice model with MI by cryoinjury and resection, GATA4 was inhibited, specifically in CMs. This inhibition downregulated FGF16, which is responsible for inhibiting fibrosis in ECM. Thus, pro-fibrotic genes (TIMP1, Col1a4) are upregulated via GATA4/FGF16 [153] (Figure 4B). Another growth factor with an anti-fibrotic function is FGF10, which has been previously analyzed in detail during the proliferation process. FGF10 acts by blocking CFs to myofibroblast differentiation, as demonstrated in mouse and human samples where high levels of FGF10 reduce collagen levels and the fibrotic process [152] (Figure 4B). On the other hand, there is a high molecular weight isoform of FGF2 (Hi-FGFG2), which is upregulated in MI and promotes myofibroblast differentiation in both in vitro and in vivo assays in mouse and human models [222].

Zhang et al. (2018) [223] identified an anti-fibrotic function for Caveolin-3 (Cav-3), since it blocks collagen deposition by inhibiting Protein kinase C (PKCε). However, low levels of Cav-3 are detected in vitro and in mice models with MI by LAD. Such decreased levels are due to an increase of miR-22 levels after cardiac injury, reducing the production of Cav-3 in CFs, which leads to an increase of collagen content, cell proliferation and promotes CFs conversion to myofibroblasts [223] (Figure 4B). AMP-activated protein kinase α1 (AMPKα1) inhibition also promotes cardiac fibrosis. AMPKα1 expression is inhibited in mice models with MI by LAD, producing an increase in CFs proliferation and myofibroblast number, whereby AMPKα exerts an anti-fibrotic effect [224] (Figure 4B). Although several anti-fibrotic proteins are overexpressed or downregulated as tools to reduce cardiac fibrosis, it is crucial to maintain a balanced situation. One example is Collagen Triple Helix Repeat Containing 1 (CTHRC1), whose levels are upregulated in mice and human fibroblasts with MI and promote fibrosis. However, in vitro assays demonstrated that an excessive downregulation of CTHRC1 produces an extreme ventricular rupture, increasing lethality. Therefore, it is important to regulate expression levels to achieve a balance in the scar and fibrosis process [225]. 

The CFs activation and myofibroblast differentiation are highly regulated processes. For instance, YAP is an upregulated protein after MI by LAD in a mice model. High levels of this protein promote myofibroblast differentiation by the interaction of YAP with TEA domain family member 1 factor (TEAD), increasing the myocardin-related transcription factor A (MRTF-A) expression and activating ECM genes expression [226]. A similar effect is produced by Fibroblast activation protein (FAP), which is also upregulated in mice and human fibroblasts with MI, although such upregulation is not detected in plasma samples [227]. Specific expression of Vestigial-like family member 3 (VGLL3) in myofibroblasts is also important to regulate the fibrotic process. After a cardiac injury, if the tissue becomes too stiff, VGLL3 is translocated to the nucleus via the β1-Rho-actin pathway, where VGLL3 binds to EWS RNA-binding protein 1 (EWSR1), inhibiting miR-29 expression. This downregulation promotes collagen deposition, increasing the fibrotic process [228]. Another protein with a pro-fibrotic effect is METTL3, a component of methyltransferase complex that regulates m6A modifications. In both in vitro (with TGF-β administration) and in vivo experiments in mice models with MI by LAD, levels of METTL3 are upregulated, promoting myofibroblast differentiation and collagen deposit [229]. RIP assays reported that METTL3 is regulated by lncRNA Metbil through the ubiquitination-proteosome process [230]. Therefore, the regulation of m6A modification mediated by Metbil/METTL3 is crucial in the fibrosis process [229,230].

As previously indicated, fibrosis development is regulated by other processes that also happen after cardiac injury, i.e., oxidative stress or inflammation [231,232,233]. Myofibroblast proliferation and differentiation are associated with oxidative stress by the Nrf2/Keap1 pathway [231] or with the inflammatory process by regulating the expression of cytokine receptors, such as Myeloid interleukin-4 receptor α (ILR4α) or Atypical chemokine receptor 4 (ACKR4) [232,233]. ACKR4 regulates IL-6 production, a pro-inflammatory cytokine that promotes fibrosis via TGF-β1/Smad/MMP2/9 [234]. After MI, an initial inflammatory response occurs, promoting macrophage recruitment, a necessary process to start fibrosis. YAP and TAZ (transcriptional coactivation with PDZ-binding motif) have been described as regulatory proteins of the inflammatory process via the Hippo pathway and also in fibrosis by regulating ECM genes [235].

The knowledge of the fibrotic process after a cardiac injury has allowed the development of several strategies to reduce this process. One of them is the use of exosomes or nanoparticles that are loaded with specific peptides, i.e., exosomes with IMTP peptide [236] with CHP [237], Sonic-Hedgehod associated with PAMs (Pharmacology active microcarriers) [238], or nanoparticles with FGF1 and NRG1 [139], reducing fibrosis in MI mice models. Another strategy is the administration of drugs or agonists of specific molecules, i.e., drugs that inhibit the canonical and non-canonical WNT pathway, inhibitors of molecules that activate stem cell proliferation, inhibitors of miRNAs with a pro-fibrotic function or molecules that inhibit the TGF-β2/Smad2/3 pathway [239,240,241,242]. There are experiments in which even a cocktail of several molecules is administered, as reported by Du et al. (2022), when five small molecules (5SM) are administered to reduce fibrosis in adult MI mice [243]. In recent years, many treatments with different types of cells have been used after cardiac injury in rodent models, i.e., NKx2-5+ cardiac progenitor cells, human mesenchymal stromal cells and endothelial colony forming cells, rat bone marrow stromal cells and hESC-CM [178,244,245,246]. In this regard, transplantation of hESC-CM for cardiac regeneration is hampered by the formation of fibrotic tissue around the grafts, preventing electrophysiological coupling of CMs [246]. However, hESC-CM survive for long periods supported by the capillary formation if hESC-CM are co-transplanted with hESC-EC [246]. Although all these strategies help to reduce the fibrotic process, the onset of fibrosis and the process of cardiac scar formation are not fully understood. Deciphering the mechanisms that regulate these events will lead to the development of new strategies for cardiac regeneration. 

## 7. The Role of Mechanical Stress in Cardiac Regeneration

Upon cardiac injury, the heart needs to start the healing process while maintaining its pumping capacity, adding, thus, an extra challenge to this process. While mechanical performance is compulsory, instead of being a hurdle, it might represent an ally. Multiple pieces of evidence have demonstrated that the maturation of stem cells into fully differentiated CMs is greatly improved by the application of mechanical forces. Currently, most of the efforts have been placed on applying distinct types of mechanical stress to different cell types, alone or in combination with distinct biocompatible scaffolds [247,248,249], with the aim of generating cardiac patches that might serve as therapeutic tools to heal the injured heart. Efforts have been made modeling distinct CMs derived from different sources, such as mesenchymal stem cells [250,251,252], cardiomyocyte progenitor cells [253,254,255,256], a mixture of cardiac cells, embryonic [257] and adult [258] CMs, induced pluripotent stem cells-derived CMs [259] and embryonic stem cell-derived CMs [260,261]. Additionally, several other studies also analyzed the functional role of mechanical load in distinct cardiovascular cells such as ECs [262] and vascular smooth muscle cells [263], as well as in a mixture of different cardiovascular cells [264,265,266,267] or ECs together with CMs [268,269], respectively. Within this context, the application of mechanical stress resulted in most cases in increased CMs differentiation, proliferation, cellular alignment as well as force production [250,253,255,257].

Insights into the molecular mechanisms driving the beneficial effects of mechanical stress are limited, involving the upregulation of key embryonic cardiac-enriched transcription factors [252,253,257,269], gap junctional proteins [268] and Wnt and Hippo signaling pathways [270]. More recently, a detailed description of the secretome resulting from mechanical conditioning of cardiac adipose tissue-derived progenitor cells was described, leading to enhanced cell adhesion, angiogenesis and immune response [271].

While in vitro evidence is highly abundant, scarce information on the modulatory effects of mechanical forces has been reported in vivo. Cassino et al. (2012) [272] reported that mechanically stimulated muscle-derived stem cells significantly improve cardiac contractility and decrease fibrosis in comparison to non-stimulated cells in a mouse model of MI, while Zimmerman et al. (2020) [273] investigated the effect of deforming mechanical loading in infarcted pig hearts, demonstrating that such deformations significantly impaired ECM and collagen orientation. To date, a single study provides in vivo evidence of the functional impact of mechanical forces during neonatal heart regeneration. Wang et al. (2022) [274] demonstrated that the natural biaxial ventricular mechanics are conserved after neonatal heart regeneration, a characteristic that is missing in adult myocardial infarcted hearts. 

## 8. Conclusions and Perspectives

Myocardial infarction is a major clinical burden worldwide and, therefore, biomedical strategies to heal the injured heart represent an unmet clinical priority [2,3]. Distinct experimental models of cardiac injury have been established, as detailed in part I of this review.

Over the last decades, new advances have been reported on the functional role of key molecular pathways modulating the cardiac injury response to hypoxia, inflammation, fibrosis, cardiomyocyte cell cycle regulation and electromechanical stimulation, as reviewed in this manuscript. However, our current understanding of the molecular pathways is still limited. Furthermore, the recent discovery of the functional role of distinct types of non-coding RNAs adds a new layer of complexity to dissect the molecular signaling pathways contributing to heal the damaged heart. Thus, additional efforts are required to decipher the complex signaling pathways modulating cardiac healing, with particular emphasis on the interactive cross talk between distinct cardiovascular cell types. In the coming years, we will, therefore, witness novel evidence on the regenerative potential of distinct cardiovascular cell types, beside cardiomyocytes, e.g., endocardial and epicardial cells, among others. However, one of the most challenging aspects in the cardiac regeneration field will be to scale and translate the cellular and molecular findings in experimental models into the clinical arena, unravelling their conservative nature and their treatment efficacy. 

## Data Availability

No new data generated.
